# Repeatability of quantitative measurements of retinal layers with SD-OCT and agreement between vertical and horizontal scan protocols in healthy eyes

**DOI:** 10.1371/journal.pone.0221466

**Published:** 2019-08-22

**Authors:** Alberto Domínguez-Vicent, Rune Brautaset, Abinaya Priya Venkataraman

**Affiliations:** Unit of Optometry, Department of Clinical Neuroscience, Karolinska Institute, Stockholm, Sweden; University of Florida, UNITED STATES

## Abstract

**Purpose:**

To evaluate the repeatability of the new spectral domain optical coherence tomography (HOCT-1F), and also to evaluate the agreement between vertical and horizontal scan protocols. In addition, we also evaluated the relation between the repeatability and age.

**Methods:**

Three consecutive measurements of the inner limiting membrane–retinal pigment epithelium (ILM-RPE), inner limiting membrane–inner plexiform layer (ILM-IPL) from macular horizontal and vertical scans, and inner limiting membrane–retinal nerve fiber layer (ILM-RNFL) from optic disc horizontal scan. 159 subjects were included in the analysis. The within subject standard deviation (Sw) and the repeatability limits (R_limit_) are used to represent the repeatability of the parameters for the different sectors.

**Results:**

The Sw for the ILM-RPE thickness was less than 3.5 μm for each sector and scan direction. The Sw values varied within the sectors and scan modes, with horizontal scan modes resulting in better values for the horizontal sectors, and vice versa. The Sw for the GCL-IPL thickness was less than 2 μm, and was similar between the vertical and horizontal scan modes for each sector map. For the optic disc scan, the Sw was not symmetric along the clock-hour map sectors, the largest Sw values were seen in the vertical sectors (8.6 μm). The mean difference between the vertical and horizontal scans was less than 2 μm for each retinal thickness sector map. Significant but weak correlation between the Sw and the subject’s age was seen in both macular and optic disc scans.

**Conclusions:**

The repeatability of the HOCT-1F to measure the ILM-RPE-, ILM-IPL- and ILM-RNFL-thickness is good. The repeatability of the ILM-RPE thickness is dependent on the scan direction, which should be taken into account when calculating retinal thickness. There is a weak correlation between the repeatability and the subject’s age.

## Introduction

Optical coherence tomography (OCT) has an irreplaceable role in the field of ophthalmology. Both clinicians and researchers use OCT to envisage and understand retinal and choroidal pathologies[1–3]. Different types of OCT are available commercially and each type comes with its own automated segmentation algorithm. The segmentation provides useful quantitative information about each individual layer of the retina. The objective measurements of macular thickness, ganglion cell-inner plexiform layer (GCL-IPL) thickness and peripapillary retinal nerve fiber layer (pRNFL) thickness provides valuable information in the diagnosis and follow up of patients with glaucoma, macular diseases and neurological disorders[4–8].

In order to know how consistent the quantitative measurements of the retinal layers are, it is important to assess the repeatability of the measurements. The repeatability of the measurement is dependent on scan resolution, acquisition time, segmentation algorithm and the retinal condition[9–13]. Current generation OCT instruments provide better resolution, faster measurements and better segmentation algorithms which in turn provides us with reliable quantitative data. The HOCT-1F (Huvitz) is a new spectral domain OCT, which has an axial resolution of around 6 to 7 microns, and acquisition rate of 68,000 A-scans per second. The three-dimensional scan can be performed with various scan resolutions and with both vertical and horizontal scan lines, both with auto- and semi-auto scanning protocols.

This study evaluates the repeatability of the HOCT-1F. We evaluated the repeatability of macular thickness, GCL-IPL thickness and pRNFL thickness. In addition, the agreement between vertical and horizontal scan protocols was also evaluated for macular thickness and GCL-IPL thickness. The relation between repeatability values with age was also evaluated.

## Materials and methods

### Study participants

The study participants were Caucasian volunteers above 18 years of age. The study adhered to the tenets of the Declaration of Helsinki and was approved by the Regional Ethical Committee (Regionala etikpröningsnämnden, Stockholm). Written informed consent was obtained after the nature and purpose of the study had been clearly explained to the participants. The study exclusion criteria were spherical refractive error more than ±5.0 D, astigmatic refractive error more than ±3.0 D, intraocular pressures more than 21 mmHg, best-corrected visual acuity worse than 0.0 logMAR for subjects under 70 years and 0.1 logMAR for subjects over 70 years of age, significant media opacities that cause poor imaging during OCT measurements, retinal disorders, and prior ocular surgeries except for cataract extraction. Participants under 70 years were excluded if they had known systemic disorders whereas no such criterion was set for participants above 70 years of age.

The subjects underwent a complete ophthalmic examination, including BCVA, intraocular pressure with non-contact tonometry, axial length measurement with optical biometer (Lenstar 900, Haag-Streit), slit lamp biomicroscopy, undilated fundus photography and OCT measurements, along with a complete ocular and medical history.

### OCT measurements

All participants underwent OCT imaging with the HOCT-1F. Only one eye (right eye) per subject was included in the analysis. A three-dimensional scan protocols composed of 512 A-scans for each of 96 B-scans was used for both macular and optic nerve head measurements. Macular scans were performed with both horizontal and vertical scanning protocols covering a 9x9 mm area centered on the fovea. For the optic nerve head scans, a horizontal scanning protocol covering 6x6 mm area centered on the optic nerve head was used. All scans were repeated 3 times with adequate breaks in between. The duration of the 9 consecutive measurements were less than 10 minutes. The scans were repeated if the fixation was poor or if the subject blinked during the measurements. Scans with signal strength less than 6 were excluded. All OCT measurements were performed by two experienced examiners. 179 subjects were recruited, and 159 subjects met the inclusion criteria and were included in the analysis.

**Fig 1:**
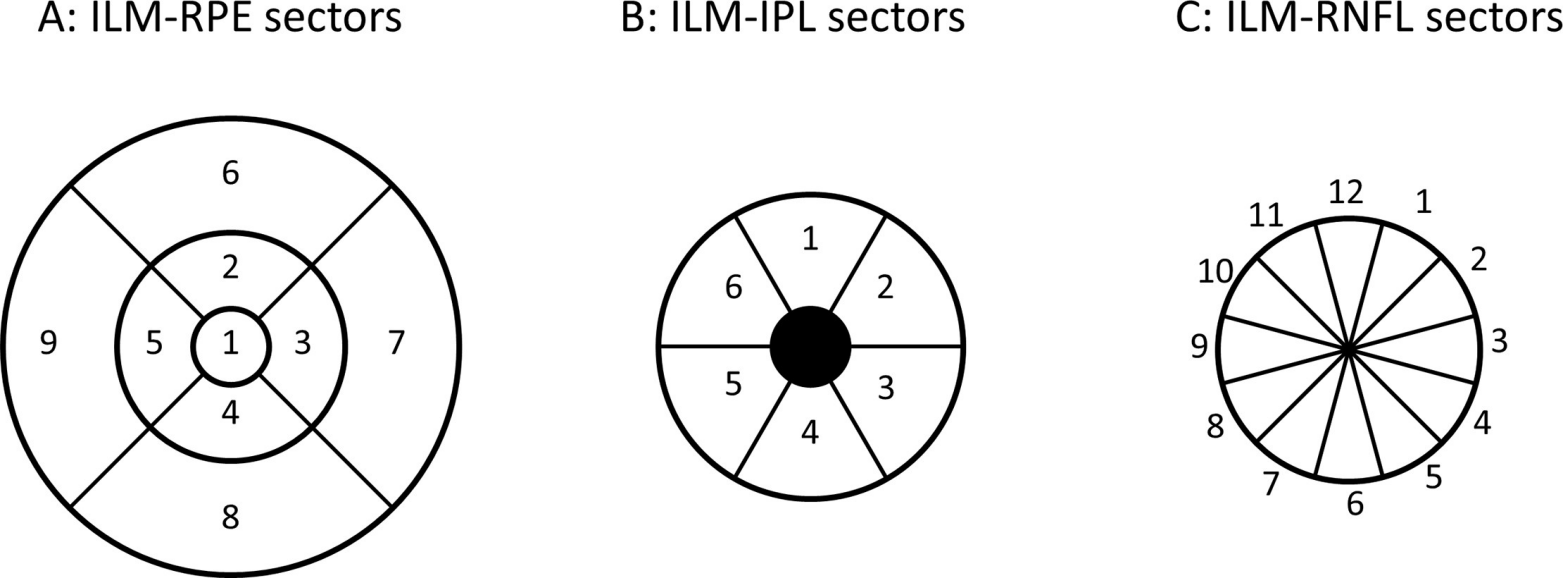
Schematic representation of the sectors for the retinal layer thickness. In A ILM-RPE represents inner limiting membrane to retinal pigment epithelium thickness and numbers 1 to 9 indicate Center, inner superior, inner nasal, inner inferior, inner temporal, outer superior, outer nasal, outer inferior and outer temporal sectors. In B, ILM-IPL represents inner limiting membrane to inner plexiform layer thickness and numbers 1to 6 indicate superior, superior nasal, inferior nasal, inferior, inferior temporal and superior temporal sectors. In C, ILM-RNFL represents inner limiting membrane to retinal nerve fiber layer thickness and the numbers represent the clock hour position of the sectors. A and B are from the macular scans and C is from the optic disc scan.

All the thickness measures were obtained using the automated segmentation algorithms from the OCT instrument. No manual adjustment of the segmentation was performed. For the macular full thickness (from inner limiting membrane to retinal pigment epithelium, ILM-RPE) evaluations, 9 sectors based on Early Treatment Diabetic Retinopathy Study (ETDRS) protocol with the central circle, inner ring (Superior, Nasal, Inferior and temporal), and outer ring (Superior, Nasal, Inferior and temporal) using 1, 3, and 6 mm diameters were used. GCL-IPL layer thickness (from inner limiting membrane to inner plexiform layer, ILM-IPL) was evaluated in 6 sectors (Superior, superior nasal, inferior nasal, inferior, inferior temporal and superior temporal) with an inner and outer circle of diameter 1, and 4 mm centered on the fovea. The pRNFL thickness (from inner limiting membrane to retinal nerve fiber layer, ILM-RNFL) was evaluated in twelve clock-hour sectors in a circle of 3.45 mm diameter centered at the optic disc. The measurement locations for the macular and the optic disc scan is shown in Fig 1.

### Statistical analysis

Descriptive statistics were used to summarize the baseline demographics of the observations and participants included. The within subject standard deviation (Sw) and repeatability limits (R_limit_) were used to describe the repeatability of the HOCT-1F to measure the ILM-RPE thickness and the ILM-IPL thickness from the macular scans, and the ILM-RNFL thickness from the optic disc scan. The Sw, which represents the repeatability of the measurements, was calculated with a one-way analysis of variance with the subject as a factor[14]. The repeatability limit was calculated as $1.96∙\sqrt{2}∙S_{w}$, and it represents the expected limits that 95% of the measurements should be within. The agreement between the horizontal and vertical scans was done with the Bland-Altman test[15] for repeated measurements for the ILM-RPE thickness and the ILM-IPL thickness from the macular scans. Finally, the Pearson correlation coefficient was used to calculate the correlation between the repeatability of the parameters and the participant’s age. The statistical significance limit was set to a p-value < 0.05.

## Results

The mean age of the 159 participants was 49.58 ± 17.01 years old [18–83 years], and the number of men and women were almost the same (77 men and 82 women). The mean axial length and spherical equivalent of the participants were 23.89 ± 0.98 mm and -0.31 ± 1.90 D, respectively. Table 1 shows the average thickness values for the ILM-RPE and GCL-IPL thickness for each sector from horizontal and vertical macular scan modalities. Within the ILM-RPE thicknesses, the thinnest value was obtained at the central sector (about 258 μm), and the thickest thicknesses were measured at the inner circle (about 320 μm) in both scan modalities. Regarding the ILM-IPL thicknesses, the temporal sectors showed about 13-μm thinner values than the other four quadrants in both scan modalities. Table 2 shows the average thickness values for the ILM-RNFL for 12 sectors from the optic disc horizontal scan. At the clock-hour map, the thicknesses among the superior and inferior sectors (clock position 11–1 and 5–7) were about 30 μm thicker than at the nasal and temporal sectors (clock position 2–4 and 8–10).

**Table 1:** Thickness values for different sectors from horizontal and vertical macular scans.

			Horizontal scan			Vertical scan		
			Average ± STD	Minimum	Maximum	Average ± STD	Minimum	Maximum
**ILM–RPE**	Central		257.5 ± 18.8	212	312	259.2 ± 19.2	212	306
	Inner circle	Superior	322.6 ± 16.2	277	364	323.8 ± 16.0	277	364
		Nasal	324.9 ± 16.2	270	363	324.6 ± 16.9	276	363
		Inferior	320.3 ± 15.5	281	364	321.5 ± 15.8	274	365
		Temporal	311.9 ± 14.8	271	353	311.9 ± 14.9	264	353
	Outer circle	Superior	273.4 ± 13.4	240	311	274.5 ± 13.6	243	318
		Nasal	295.6 ± 16.6	258	338	294.6 ± 16.1	255	336
		Inferior	265.2 ± 14.3	229	311	267.0 ± 13.8	231	310
		Temporal	259.5 ± 13.5	221	301	259.3 ± 14.0	223	302
**ILM–IPL**	Superior		116.5 ± 9.7	83	143	117.7 ± 9.7	81	144
	Nasal superior		119.5 ± 9.7	85	144	119.5 ± 9.8	82	144
	Nasal inferior		119.0 ± 10.4	81	143	119.2 ± 10.4	82	142
	Inferior		116.3 ± 9.5	89	142	117.7 ± 9.7	89	144
	Temporal inferior		106.3 ± 8.3	80	129	107.1 ± 8.5	79	129
	Temporal superior		103.3 ± 8.0	75	126	104.0 ± 8.3	74	126

ILM-RPE: Inner limiting membrane to retinal pigment epithelium ILM-IPL: Inner limiting membrane to inner plexiform layer, Ganglion cell-inner plexiform layer.

STD: Standard deviation.

All values are expressed in microns.

**Table 2:** Retinal nerve fibre layer thickness values for different sectors from horizontal optic disc scans.

Sector	Average ± STD	Minimum	Maximum
12	110.9 ± 23.4	39	184
1	101.6 ± 20.5	38	173
2	97.2 ± 21.3	38	145
3	65.9 ± 14.8	33	109
4	77.6 ± 15.7	28	126
5	100.5 ± 19.2	28	154
6	127.3 ± 21.5	59	184
7	133.3 ± 18.1	83	180
8	75.7 ± 14.3	40	119
9	59.6 ± 8.81	37	102
10	85.6 ± 14.5	43	164
11	130.4 ± 19.6	56	172

STD: Standard deviation.

All values are expressed in microns.

### Intra-device repeatability

**Fig 2:**
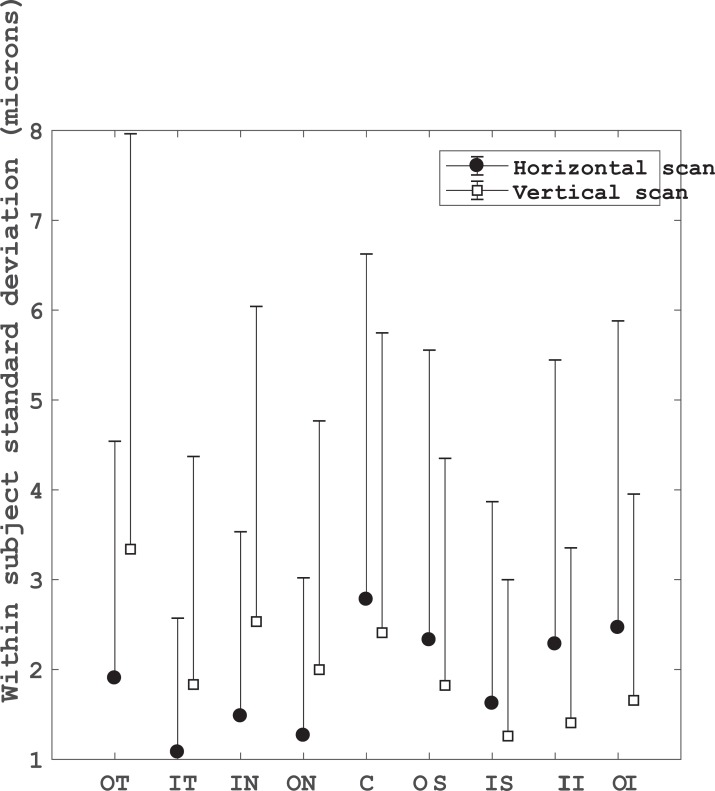
Intra-device repeatability for the inner limiting membrane to retinal pigment epithelium thickness in different sectors for horizontal and vertical scans. OT: outer temporal, IT: inner temporal, IN: inner nasal, ON: outer nasal, C: central circle, OS: outer superior, IS: inner superior, II: inner inferior, OI: outer inferior sectors.

The intra-device repeatability of three consecutive measurements of the ILM-RPE thickness is shown in Fig 2. Overall, the repeatability of the measurements for the ILM-RPE thickness map was less than 3.5 μm for each sector and scan direction. The Sw for the Nasal and Temporal sectors was about 0.8 and 1.5 μm less with the horizontal scan mode than with the vertical one. Furthermore, the smallest R_limits_ were obtained with the horizontal scan mode, which was about 2.5 μm less than the vertical scan mode. The opposite tendency was obtained for the Superior and Inferior sectors, in which the Sw and R_limit_ values with the Horizontal scan mode were about 1- and 2 μm more than the Vertical mode, respectively. Finally, the Sw for the central sector was 2.8 and 2.4 μm with the Horizontal and Vertical scan modes, respectively. The R_limit_ for this sector was about 7 μm for both scan modes.

**Fig 3:**
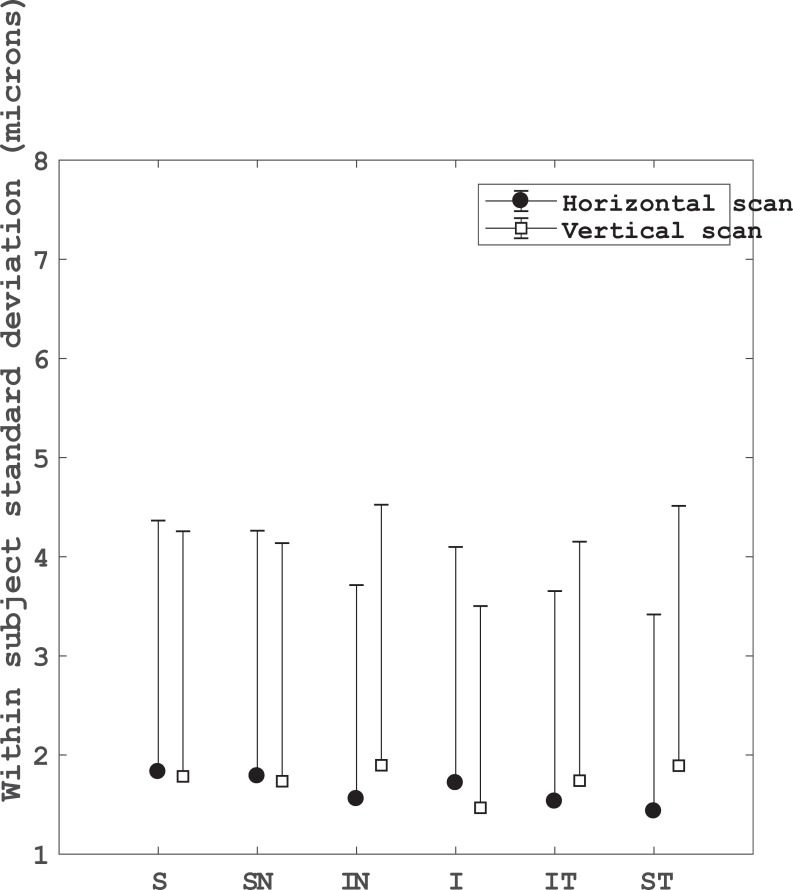
Intra-device repeatability for the inner limiting membrane to inner plexiform layer thickness in different sectors for horizontal and vertical scans. S: superior, SN: superior nasal, IN: inferior nasal, I: inferior, IT: inferior temporal and ST: superior temporal sectors.

The intra-device repeatability of three consecutive measurements of the GCL-IPL thickness is shown in Fig 3. The Sw and R_limit_ were similar between the vertical and horizontal scan modes for each sector map. The Sw ranged from 1.4 to 1.9 μm, and the R_limit_ ranged from 3.9 to 5.3 μm.

**Fig 4:**
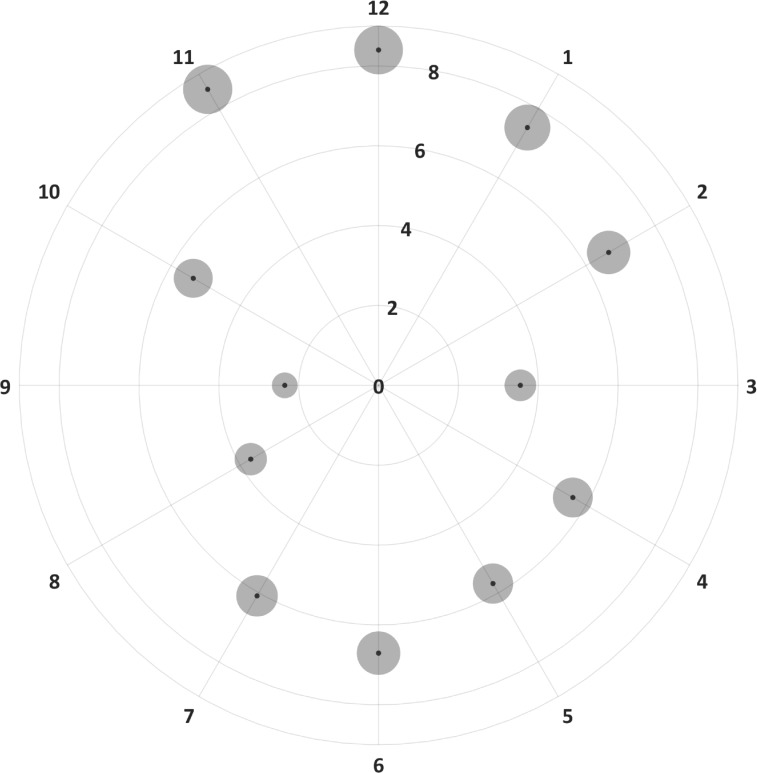
Intra-device repeatability for the inner limiting membrane to retinal nerve fiber layer thickness in different clock hour sectors for horizontal scans. The small black dots represent the within subject standard deviation and the grey circles surrounding each black point represent the repeatability limits, where the areas are scaled by a factor of 30 points for visualization purposes.

Fig 4 shows a polar plot with the repeatability of the three measurements obtained for the RNFL thickness for each clock-hour sector. In this plot, the central small black points represent the Sw, and the grey circles surrounding each black point represents the R_limit_ of each Sw. The size of these circles was scaled for visualization purpose to facilitate the comparison of the R_limit_ among sectors. Overall, the repeatability was not symmetric along the clock-hour map sectors. As it can be seen, the repeatability was low for the vertical sectors, and the best repeatability values were obtained for the horizontal sectors. Concretely, the Sw at the 12- and 6 clock-hour sectors were above 6.5 μm, whereas it is as low as 2.3 μm on the 9 clock-hour sector. Similarly, the R_limit_ was also not symmetric, with minimum values at the horizontal sectors and maximum values at the vertical ones. Concretely, the maximum R_limit_ was obtained at the 12 clock-hour sector (23.3 μm), and the minimum R_limit_ was obtained at the 9 clock-hour sector (6.5 μm).

### Agreement between horizontal and vertical scans

Table 3 shows the agreement between the horizontal and vertical scans for the ILM-RPE and ILM-IPL thicknesses. The mean difference between the vertical and horizontal scans was less than 2 μm for each retinal thickness sector map. The mean difference for the horizontal sectors (Nasal and Temporal) was about 1 μm less than for the vertical ones (Superior and Inferior). Nevertheless, the maximum variability between the horizontal and vertical scan directions was between 9 μm and 17 μm for the inner and outer circle sectors. The vertical scan measured on average 1.67 μm thinner than the horizontal scan in the central sector, and the maximum variability between both scan directions was 20.13 μm.

**Table 3:** Comparison between the horizontal and vertical macular scan.

	Map sectors		Mean difference [Limits of Agreement]
**ILM–RPE**	Central		-1.67 [-11.77–8.43]
	Inner circle	Superior	-1.24 [-6.27–3.78]
		Nasal	0.25 [-7.76–8.26]
		Inferior	-1.23 [-9.09–6.63]
		Temporal	-0.03 [-4.81–4.75]
	Outer circle	Superior	-1.07 [-8.25–6.10]
		Nasal	0.97 [-5.78–7.73]
		Inferior	-1.83 [-8.65–4.98]
		Temporal	0.24 [-8.40–8.88]
**ILM–IPL**	Superior		-1.27 [-6.92–4.38]
	Nasal superior		0.00 [-5.65–5.64]
	Nasal inferior		-0.20 [-6.33–5.94]
	Inferior		-1.39 [-6.61–3.83]
	Temporal inferior		-0.81 [-5.92–4.31]
	Temporal superior		-0.64 [-5.83–4.55]

ILM-RPE: Inner limiting membrane to retinal pigment epithelium.

ILM-IPL: Inner limiting membrane to inner plexiform layer.

All values are expressed in microns.

With regard to the ILM-IPL thickness, the mean difference between the vertical and horizontal scans was less 2 μm for each sector. The mean differences for the horizontal sectors (Nasal superior, Nasal inferior, Temporal superior, and Temporal inferior) was less than for the vertical sectors (Superior and Inferior). Considering the limits of agreement, the maximum difference between both scan directions could be up to 12.3 μm.

### Repeatability as a function of the subject’s age

Tables 4 and 5 show the correlation values between the Sw and the participant’s age for the macular and optic disc maps, respectively. Although the correlations between the Sw and the age were statistically significant (P < 0.05) for some sectors, the correlation was weak in all sectors for both macular and optic disc maps.

**Table 4:** Correlation values between the repeatability and age for the macular scans.

			Horizontal scan	Vertical scan
**ILM–RPE**	Central		0.251*	0.010
	Inner circle	Superior	0.127	-0.015
		Nasal	0.029	0.004
		Inferior	0.068	0.059
		Temporal	0.056	0.153
	Outer circle	Superior	0.138	-0.016
		Nasal	0.187	0.158*
		Inferior	0.145	0.012
		Temporal	0.162	0.174*
**ILM–IPL**	Superior		0.250*	0.033
	Nasal superior		0.216*	0.082
	Nasal inferior		0.061	0.007
	Inferior		0.243*	0.178*
	Temporal inferior		0.076	0.052
	Temporal superior		0.135	0.059

ILM-RPE: Inner limiting membrane to retinal pigment epithelium

ILM-IPL: Inner limiting membrane to inner plexiform layer, Ganglion cell-inner plexiform layer.

* Indicates p<0.05

**Table 5:** Correlation values between the retinal nerve fibre layer thickness repeatability and age for the optic disc scans.

Sectors	Horizontal scan
**12**	0.161*
**1**	0.316
**2**	0.178*
**3**	0.121
**4**	0.340*
**5**	0.192*
**6**	0.325*
**7**	0.222*
**8**	0.093
**9**	0.078
**10**	0.301*
**11**	0.300*

* Indicates p<0.05

## Discussion

The aim of this study was to assess the repeatability of the Huvitz HOCT-1F system to measure the retinal-, GCL/IPL, and RNFL thicknesses. Based on the results of this study, clinicians will be able to set the protocols for OCT scans.

The intra-device repeatability of each ILM-RPE sector map (Fig 2) varied with the scan direction (Horizontal or Vertical). The best repeatability outcomes for the horizontal sectors (Nasal and Temporal) were obtained with the horizontal scan direction. On the other hand, the best repeatability values for the vertical sectors (Superior and Inferior) were obtained with the vertical scan direction. Whereas, the repeatability for the central sector was similar with both scan directions. The maximum Sw and R_limit_ (3.339 and 9.248 μm, respectively) were seen for the temporal sector in the outer circle from the vertical scan. Nevertheless, the maximum Sw reported here is less than the axial resolution (6–7 μm) of the HOCT-1F instrument. The R_limit_ was also less than the axial resolution of the instrument in 5 out of 9 sectors for the horizontal scan, and 6 out of 9 sectors for the vertical scan. The intra-device repeatability of each ILM-IPL sector map (Fig 3) was similar for both scan direction (Horizontal or Vertical). Both Sw and R_limit_ were less than the axial resolution of the instrument for the same measurement conditions used this study.

Several previous studies have also reported good repeatability for both ILM-RPE and ILM-IPL [11,13,16–19]. All in all, most of these studies assessed the repeatability of different SD-OCT to measure the GCL/IPL and RNFL thicknesses. Some of the studies reported the repeatability using the intraclass correlation coefficient, which values were greater than 0.9 for the GCL/IPL and greater than 0.8 for the RNFL in each sector thickness map [13,17,18]. Another study described the repeatability of six SD-OCTs to measure the central retinal thickness using the coefficient of variation, and this value ranged from 0.46 to 3.50 [16]. Finally, Ctori et al. assessed the repeatability of a SD-OCT to measure each retinal layer, and the coefficient of repeatability ranged from 0.31 (retinal thickness) to 1.73 (outer nuclear layer) [19]. Since different metrics have been used to describe the repeatability of a specific SD-OCT, direct comparisons among studies are difficult to make. Besides all this, it should be taken into account that these results evidence that the repeatability of the automatic segmentation algorithms available in the SD-OCT is good.

**Fig 5:**
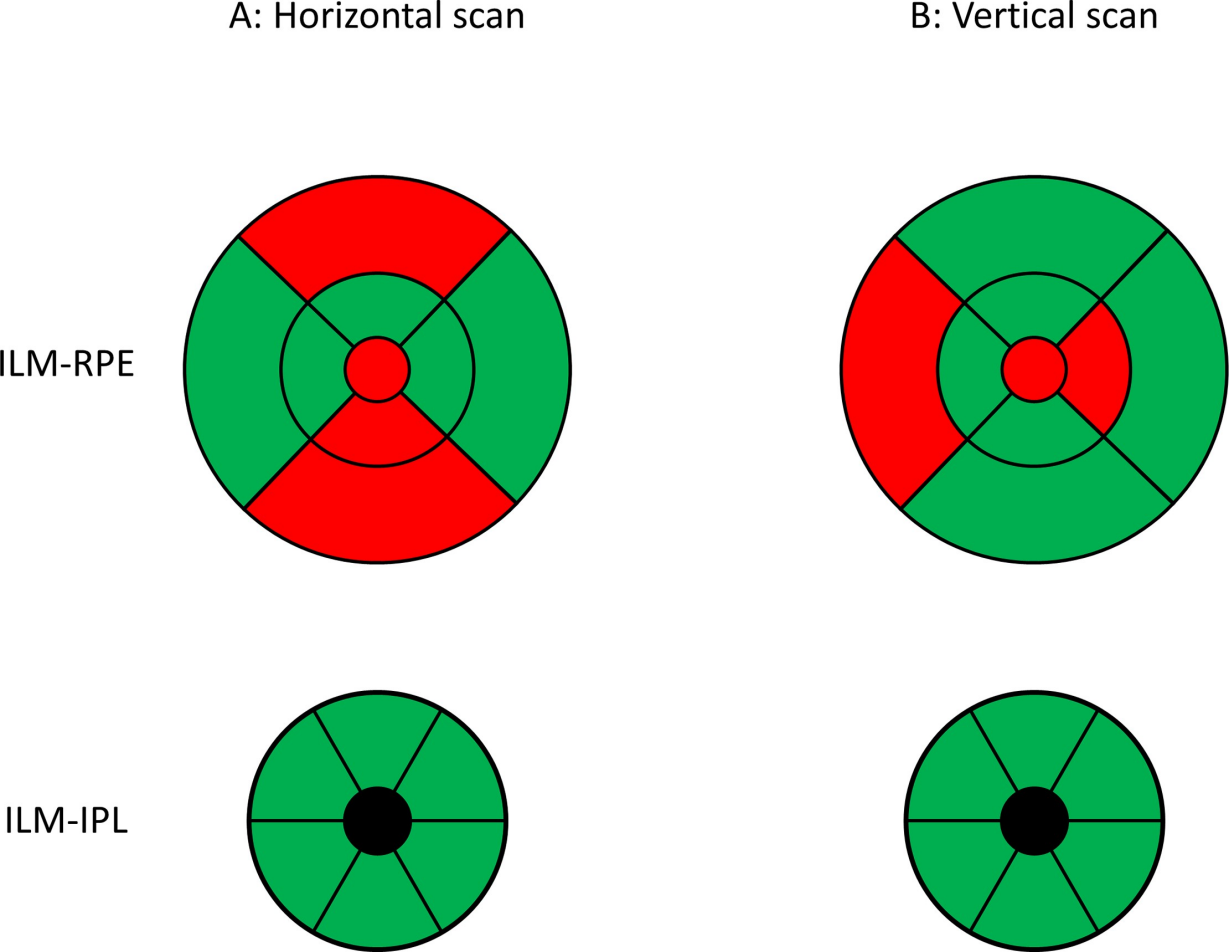
Colour coded map of the intra-device repeatability for the macula scans in different sectors. Green: repeatability limit values within the instrument’s axial resolution, Red: repeatability limit values above the instrument’s axial resolution. ILM-RPE: inner limiting membrane to retinal pigment epithelium thickness ILM-IPL: inner limiting membrane to inner plexiform layer thickness.

Fig 5 show a colour coded map of the intra-device repeatability for the macula scans, where R_limit_ values more than the instrument’s axial resolution are marked in red. A previous study reported that there is no variation in the repeatability values between horizontal and vertical scans based on analyses from 20 healthy eyes and 20 eyes of patients with multiple sclerosis[20]. However, that study did not evaluate sector wise the repeatability as it is done in the present study. We believe that the variation in the repeatability can be seen in a sector wise analysis rather than the analysis of the whole scanned area. This variation could be a result of the influence of the retinal blood vessel in the segmentation algorithm. The B-scan used for the calculation of Nasal and Temporal sectors could pass through less number of blood vessels in horizontal scan mode compared to the B-scan from the vertical scan mode, and vice versa for the vertical sectors. From our results, it can be recommended to use vertical scans for vertical sectors, and horizontal scans for horizontal sectors. In other words, the accuracy of the quantitative measurements with OCT can be improved by performing both horizontal and vertical scans consecutively, and combining the information from both scans in the calculations of the retinal thickness.

The intra-device repeatability of the ILM-RNFL from the optic disc scan varied with the clock-hour sector (Fig 4). The repeatability was worse for the vertical sectors compared to the horizontal sectors. In comparison to the repeatability of the parameters from the macular scans, the optic disc scan showed lower repeatability as shown by larger Sw. This finding is not surprising as the influence of the blood vessels are more in vertical clock-hour positions compared to the horizontal clock-hour positions[21–23]. Previous studies have also reported the same trend.

With the agreement analysis, it can be seen that on average, the difference between horizontal and vertical scans is minimal for both ILM-RPE and ILM-IPL thicknesses (Table 3). However, large variabilities in the differences are seen in the ILM-RPE thickness in the outer circle and centre. The variabilities in the differences are similar between the ILM-RPE thickness in the inner-circle and the ILM-IPL thickness. It is almost the same retinal location that it is used in the analysis of the inner circle of the ILM-RPE and ILM-IPL. This area does not have that many blood vessels as the outer circle of the ILM-RPE analysis. This reasoning does not explain the large variability in the difference of the central ILM-RPE thickness as there are no blood vessels in the centre. The possible reason for this could be that the central values are critically dependent on the scan centration[24].

The retina undergoes significant changes as a part of the normal aging process[25,26]. It would be interesting to know how the repeatability of OCT parameters varies with age. There is a significant correlation between the repeatability and the subject’s age in some of the sectors of the macular and optic disc scans (Tables 4 and 5), however the correlation coefficient values were weak (between 0.15 and 0.34). Liu et al. showed that age is not one of the factors that increase the prevalence of artefacts in OCT imaging[27]. However, it could be difficult for older subjects to fixate during OCT measurements, but the faster acquisition time and better scan algorithms make it possible to have good repeatability independent of the subject’s age.

According to the maximum Sw that we got for the ILM-RPE, ILM-IPL, and ILM-RNFL thicknesses, we calculated the measurement tolerance, MT (*MT* = (1.96∙*Sw*)/√*N*) for N number of measurements according to the ISO standards[28,29]. For 1, 2, and 3 number of measurements, the measurement tolerance for the ILM-RPE is 6.54, 4.63, and 3.78 μm, respectively. For the ILM-IPL, the measurement tolerances were 3.72, 2.63, and 2.15 μm, respectively. For the ILM-RNFL from the optic disc scan, the measurement tolerances were 16.77, 11.86, and 9.68 μm, respectively. For the macula thickness measurements in retinas with no morphological changes, one three-dimensional scan protocol composed of 512 A-scans for each of 96 B-scans is enough to have a tolerance within the axial resolution of the instrument. However, when it comes to the RNFL thickness from the peripapillary scans, more than 3 scans with the above set parameters are needed to ensure a tolerance within the axial resolution of the instrument. However, the number of scans required to achieve these tolerance limits can vary for retinas with morphological changes. Further studies can also consider the repeatability analyses of different scans protocols, in both healthy as well as pathological retinas.

A previous study has shown that the repeatability of automatic segmentation algorithms in pathologic eyes is worse than in normal eyes [30]. However, the repeatability improved up to the level of healthy eyes when manual corrections of the segmentation were performed. As a consequence, the intrinsic variability in manual measurements might affect the reproducibility of the measurements in pathologic eyes. The present study included only healthy eyes to assess the performance of HOCT-1F but further studies are recommended to assess the performance in pathologic eyes. On the other hand, several studies reported a positive relationship between the signal strength and the pRNFL thickness [31,32]. This relationship indicates that low signal strength is associated with thin pRNFL thickness. As the present study included scans with a good signal strength only, further studies can be performed to assess the correlation between the signal strength and the repeatability.

In conclusion, the repeatability of the Huvitz HOCT-1F to measure the ILM-RPE thickness, ILM-IPL thickness and ILM-RNFL thickness is good. The repeatability of the ILM-RPE thickness is dependent on the scan direction, and this factor should be taken into account while choosing scan protocols. A significant but weak correlation is seen between the repeatability of the HOCT-1F and the subject’s age.

## Supporting information

S1 File RawData. (XLS)
